# The Role and the Regulation of NLRP3 Inflammasome in Irritable Bowel Syndrome: A Narrative Review

**DOI:** 10.3390/microorganisms13010171

**Published:** 2025-01-15

**Authors:** Arezina Kasti, Konstantinos Katsas, Maroulla D. Nikolaki, Konstantinos Triantafyllou

**Affiliations:** 1 Department of Nutrition and Dietetics, Attikon University General Hospital, 12462 Athens, Greece; kastiare@med.uoa.gr (A.K.); katkonstantinos@gmail.com (K.K.); maroullanikolaki@gmail.com (M.D.N.); 2 Hepatogastroenterology Unit, 2nd Department of Internal Propaedeutic Medicine, Medical School, National and Kapodistrian University of Athens, Attikon University General Hospital, 12462 Athens, Greece

**Keywords:** irritable bowel syndrome, NLRP3 inflammasome, visceral hypersensitivity, proinflammatory cytokines

## Abstract

Irritable bowel syndrome (IBS) is a chronic disorder of the gastrointestinal tract. Its pathogenesis involves multiple factors, including visceral hypersensitivity and immune activation. NLRP3 inflammasome is part of the nucleotide-binding oligomerization domain-like receptor (NLR) family, a crucial component of the innate immune system. Preclinical studies have demonstrated that inhibiting NLRP3 reduces visceral sensitivity and IBS symptoms, like abdominal pain, and diarrhea, suggesting that targeting the NLRP3 might represent a novel therapeutic approach for IBS. This review aims to assess the NLRP3 inhibitors (tranilast, β-hydroxybutyrate, Chang-Kang-fang, paeoniflorin, coptisine, BAY 11-7082, and *Bifidobacterium longum)*, highlighting the signaling pathways, and their potential role in IBS symptoms management was assessed. Although premature, knowledge of the action of synthetic small molecules, phytochemicals, organic compounds, and probiotics might make NLRP3 a new therapeutic target in the quiver of physicians’ therapeutic choices for IBS symptoms management.

## 1. Introduction

The prevalence of irritable bowel syndrome (IBS) varies throughout the world. Globally, approximately 10–20% of the population suffers from IBS [1].

Cases of IBS have increased in recent years, resulting in higher healthcare utilization. IBS is a disorder of gut–brain interaction (DGBI) characterized by recurring and intermittent gastrointestinal dysfunction [2], including abdominal pain associated with altered bowel habits [2,3,4]. IBS pathogenesis includes chronic, low-grade, inflammation implicated in the disease process perpetuating the symptoms of IBS [5]. IBS treatment involves adjusting eating habits and other lifestyle changes, medicines, probiotics, and mental health therapies. Corticotropin-releasing factor (CRF) modulates gastrointestinal alterations and promotes. It promotes inflammation, which is present in DGBIs, by stimulating the release of proinflammatory cytokines, Tumor necrosis factor-alpha (TNF-α), interleukin (IL) IL-1, and interleukin (IL) IL-6. An impaired gut barrier is vital in the pathogenesis of IBS. A leaky gut allows bacterial translocation, triggering the immune system to produce lipopolysaccharide (LPS) and proinflammatory cytokines, which can cause visceral hypersensitivity and worsen gut barrier function [6,7].

NOD-like receptors (NLRs) are a family of cytosolic pattern recognition receptors (PRRs) implicated in the innate immune sensing of pathogens and damage signals. NLRs act as sensors in multi-protein complexes called inflammasomes. Inflammasome activity is necessary for the maintaining of intestinal homeostasis, although their aberrant activation contributes to the pathogenesis of several gastrointestinal diseases. Inflammasomes serve as innate immune sensors, activating inflammatory responses [8]. They recognize signals, activate caspase-1, produce IL-1β and IL-18, and initiate the inflammatory process. NLR proteins contain a central NACHT nucleotide-binding domain (NBD) and carboxy-terminal leucine-rich repeats (LRRs) but differ in terms of their N-terminal since the NLRC proteins have one or more caspase recruitment (CARD) domains whereas the NLRP proteins have pyrin domains (PYD) at their N-terminal [9]. The NLRP3 inflammasome plays a role in various inflammatory diseases, metabolic disorders, autoimmune diseases, and cancer. In the gut, NLRP3 plays a crucial role in host defense, regulating intestinal homeostasis, maintaining the integrity of the epithelium, and modulating immune responses associated with the microbiota. Other well-studied inflammasomes like NLRP1 were associated with autoimmune disorders affecting the skin, lungs, arthritis, neurodegenerative diseases, and cancer. The NLRC4 is activated in response to bacterial infections and relies on specific bacterial protein recognition by NLR proteins. NLRP6 is crucial for intestinal homeostasis, host defense in epithelial cells, and innate immune signaling in myeloid cells; its deficiency hindered mucin secretion by goblet cells, raising infection susceptibility [9,10].

Therapeutic discoveries target the inflammasome pathway, highlighting promising novel strategies in intestinal disease treatments. Collectively, our understanding of the mechanisms of intestinal inflammasome activation and their interactions with other immune pathways appears to be not fully elucidated. Moreover, the clinical relevance of the efficacy of inflammasome inhibitors has not been evaluated. Despite these limitations, a greater understanding of the effectiveness, specificity, and reliability of pharmacological and natural inhibitors that target inflammasome components could be an opportunity to develop new therapeutic options for intestinal disease treatments [9].

**Figure 1:**
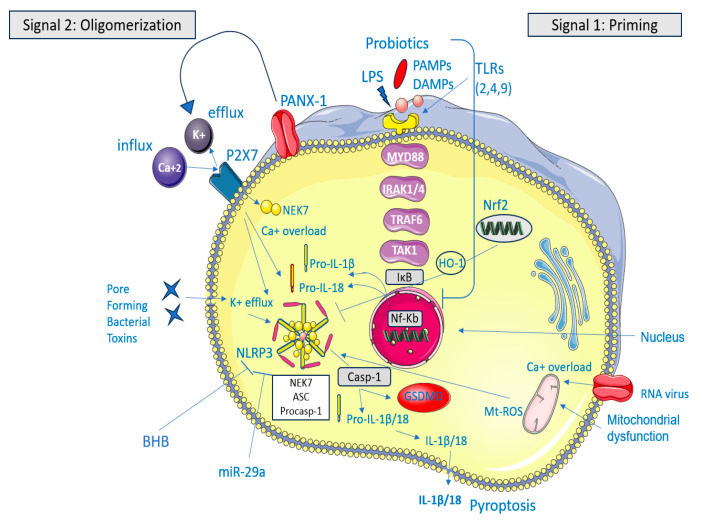
The NLRP3 inflammasome acts as a sensor–adaptor–effector system that detects cell stress and membrane damage. Normally, NLRP3 levels are insufficient to activate myeloid immune cells, keeping ASC and caspase-1 stable [8]. Upon activation, ASC and caspase-1 form a complex with NLRP3, activating caspase-1, which then converts pro-inflammatory cytokines IL-1β and IL-18 into their active forms, triggering an inflammatory response [5]. Canonical activation of the NLRP3 inflammasome involves two signals: priming (Signal 1), which increases levels of its components in response to PAMPs or DAMPs, and oligomerization (Signal 2), which occurs due to stress signals like ion disturbances and mitochondrial reactive oxygen species. The activation of NLRP3 results in its aggregation with ASC and pro-caspase-1, which leads to the maturation of interleukin-1 beta (IL-1β) and interleukin-18 (IL-18) as well as the cleavage of gasdermin D (GSDMD). This cleavage triggers pyroptosis. In IBS, PAMPs are considered the dysbiotic gut microbiota (and the bacterial toxins) that trigger NLRP3 activation [9]. The figure was designed using Servier Medical Art, provided by Servier, licensed under a Creative Commons Attribution 3.0 unported license.

Patients with IBS have altered NLRP3 expression that exacerbates intestinal inflammation, attracting inflammatory cells like macrophages, monocytes, and neutrophils, which produce reactive oxygen species (ROS) [8], as shown in Figure 1.

NLRP3 deficiency decreases intestinal inflammation, highlighting that normal IL-1β secretion is critical for this condition [11]. Traditionally, IBS has been a disorder with no known underlying structural or biochemical explanation, but this concept is likely to be outdated. Underlying mechanisms that could lead to irritable bowel syndrome include genetic factors (most notably an identified mutation of SCN5A); post-infectious changes, chronic infections and disturbances in the intestinal microbiota; low-grade mucosal inflammation, immune activation, and altered intestinal permeability; disordered bile salt metabolism (in 10–20% of cases with diarrhea); abnormalities in serotonin metabolism; and alterations in brain function, which could be primary or secondary factors. Identical IBS symptoms are probably due to different disease processes; grouping patients with this disorder into either diarrhea-predominant or constipation-predominant subtypes promotes heterogeneity. An approach based on the underlying pathophysiology could help develop therapies that target causes and ultimately provide a cure for patients with IBS [11]. The NF-κB and Mitogen-Activated Protein Kinase (MAPK) signaling pathways are integral to the pathogenesis of a wide range of inflammatory diseases, including IBS among others, making them potential therapeutic targets for inflammation [12]. Researchers also exploring methods to inhibit the NLRP3 by targeting its complex signaling pathways, including activation priming, microenvironment ion levels (K^+^, Ca^2+^, Cl^−^, and ROS), inflammasome assembly, and GSDMD cleavage [13]. Scientific evidence has demonstrated that increased IL-1β levels are linked to IBS and NLRP3 activation [11,12,14].

To date, there have been few preclinical studies on NLRP3 modulation in IBS, but no experiments have been conducted in human subjects. This review evaluates NLRP3 inhibitors in preclinical studies, focusing on these signaling pathways and their impact on IBS symptoms. The effectiveness of specific NLRP3 inflammasome inhibitors [Tranilast (INN, brand name Rizaben, Kissei Pharmaceuticals Japan and South Korea), β-hydroxybutyrate, Chang-Kang-fang, paeoniflorin, coptisine, BAY 11-7082 (Calbiochem, Germany) and *Bifidobacterium longum* will be assessed.

## 2. Intestinal Inflammation and IBS

IBS is characterized by dysbiosis of the gut microbiota and chronic low-grade inflammation, triggered by the accumulation of commensal bacteria in the intestinal lumen. Low-grade mucosal inflammation may account for visceral hypersensitivity [14]. Histopathological studies in IBS patients revealed elevated levels of pro-inflammatory cytokines and mast cells (MCs) important in the innate immune response. Degranulated MCs near nerve fibers in the mucosa release inflammatory mediators and activate smooth muscle cells and enteric neural activity [11,14,15]. In the diarrhea subtype of IBS, MC activation and infiltration are linked to visceral pain and increased T-lymphocytes [16]. Histological analysis also shows intestinal inflammation and elevated cytokine levels in peripheral blood mononuclear cells (PBMCs) [17].

In a mouse model of visceral hypersensitivity, supernatants from healthy controls inhibited colonic afferent nerve endings more effectively than those from IBS patients. Additionally, lower β-endorphin concentrations in PBMCs derived from IBS patients contributed to reduced inhibitory effects, indicating immune function changes may play a role in visceral hypersensitivity in IBS [18].

Analysis of supernatants from IBS-D patients revealed increased levels of IL-1β, IL-6, IL-10, and TNFα, correlating with pain frequency and severity [19]. Patients with post-infectious irritable bowel syndrome (PI-IBS) show increased intestinal permeability, as indicated by urinary excretion of lactulose and mannitol [20]. Similarly, in non-infectious IBS patients, abnormal intestinal permeability and reduced zonulin protein (ZO-1) levels have been observed [11,21,22], and confocal laser endomicroscopy reveals significant epithelial gaps in the terminal ileum of IBS patients compared to healthy controls [11,23]. There are also disturbances in tight junction proteins like ZO-1, claudin-1, and junctional adhesion molecules in IBS patients [11,24], with MC degranulation leading to decreased expression of these proteins, likely due to tryptase release [11,25]. Grabauskas et al. found that colon biopsies from IBS-D patients have high levels of prostaglandin E2 (PGE2) and increased cyclooxygenase-2 (COX-2) gene expression [26]. Additionally, IBS patients often display dysregulation of NLRP3, with heightened IL-1β and caspase-1 levels, contributing to mucosal inflammation [27].

## 3. Potential Inhibitors of NLRP3 in IBS

Table 1 summarizes evidence from preclinical studies showing that inhibiting NLRP3 may reduce cytokines and other proinflammatory protein production, suggesting a novel therapeutic target for IBS via regulation of inflammation.

### 3.1. Phytochemicals

#### 3.1.1. Paeoniflorin Through miR-29a

MiRNAs are short non-coding RNA molecules that regulate gene expression by binding to target mRNAs, causing a block in translation and mRNA degradation [32]. Intestinal microRNAs serve as regulators at the host–microbial interface. Exosomes containing specific microRNA sequences derived from intestinal epithelial cells (IECs) were detected in the stools of mice. Transgenic mice unable to produce IECs-derived miRNA showed an increased luminal content of Firmicutes and Proteobacteria phyla and variations of specific bacterial families. Additionally, chemical-induced inflammation altered the miRNA profile in mouse models, confirming the importance of IEC as a primary source of miRNA in the gut, while an altered miRNA profile has been related to intestinal barrier dysfunctions in IBS-D patients [33]. miRNA-29a regulates intestinal membrane permeability via the glutamine synthetase gene in IBS-D [34]. Over-expression of miR-29a significantly enhanced epithelial permeability in IBS [32] and regulated NLRP3 expression at the post-transcriptional level by complementary binding to their target NLRP3 gene [35].

Paeoniflorin (PF) sulfonate is a monoterpene glycoside with formula C23H28O14S, originally isolated from the roots of Paeonia lactiflora. PF has been traditionally used to treat pain and immunologic disturbances in China [36]. Ke et al. investigated how this bioactive component alleviates the inflammatory response in a mouse model of IBS-D. C57BL/6 wild type (WT) and miR-29a knockout (KO) mice were randomly divided into four groups, with seven per group: (rifaximin, 100 mg/kg; PF 50 mg/kg; the control; and the IBS-D groups, respectively). Visceral sensitivity in the PF group was lower than in the model group, both WT and miR-29a KO mice. In both lineage mice, damage was observed in the colon tissues of the IBS-D group, while PF partially improved the tissue damage. Serum levels of IL-1β, IL-18, TNF-α, and MPO decreased in the PF group; being more prominent in the miR-29a KO mice compared with WT mice. The expression of NLRP3 was lower in miR-29a mice suggesting that PF may inhibit the NLRP3 inflammasome pathway by downregulating miR-29a expression [29].

#### 3.1.2. Coptisine Through Nrf2 Signaling Pathway

Transcription factor nuclear factor erythroid 2-related factor 2 (Nrf2) is a key regulator of oxidative stress and ROS generation. Nrf2 is a basic leucine zipper protein that under normal conditions is in the cytoplasm. Upon activation, the movement of Nrf2 into the cell nucleus depends on the balance of nuclear import and export signals. A redox-sensitive signal causes Nrf2 to accumulate in the nucleus by preventing the interaction between nuclear exportin and nuclear export sequences. One of the main functions of Nrf2 is to promote the body’s antioxidant response, making it a crucial tool for cell survival. The NLRP3 and Nrf2 signaling pathways are linked by their response to oxidative stress and ROS formation. Nrf2 inhibits pro-inflammatory cytokines and regulates NF-κB activity, while NF-κB releases secondary inflammatory mediators such as COX-2 [35].

It is well documented that oxidative stress and inflammation are involved in the pathophysiology of post-infectious IBS (PI–IBS) [30], while the activation of the Nrf2 signaling pathway protects from PI–IBS in in vivo and in vitro models [37]. Additionally, evidence suggests that neuroinflammation may impair the enteric nervous system (ENS), leading to increased sensitivity and disrupted intestinal peristalsis. This could be due to an exaggerated response from immune cells, such as MCs and enterochromaffin cells in the colon, which signal inflammation to the ENS and trigger the release of serotonin and other cytokines, thus promoting the local production of ROS [38]. Excessive ROS may activate inflammatory signaling pathways like NF-κB and the NLRP3 [39]. Excess of ROS causes thioredoxin (TRX) dissociation from thioredoxin-interacting protein (TXNIP), leading to TXNIP binding to NLRP3 and initiating inflammasome activation [40].

Coptisine (COP) is the active ingredient of Rhizoma Coptidis, an Asian phytomedicine with anti-inflammatory [41,42], antimicrobial, and antioxidant action [43]. Xiong et al. studied the protective effects of COP against PI-IBS by inhibiting the NLRP3 through the Nrf2 signaling pathway in rats. Sprague–Dawley rats were randomly divided into four groups (n = 20 per group): (i) Control; (ii) PI–IBS model; (iii) COP treatment (50 mg/kg); and (iv) COP (50 mg/kg) + ML385, an Nrf2 transcription factor inhibitor, (30 mg/kg) treatment. Treating rats with COP significantly increased the protein expression of nuclear Nrf2 and significantly decreased cytoplasmic Nrf2 expression compared to the PI–IBS group. Additionally, the protein expression levels of NLRP3 and ASC were markedly higher in the PI–IBS group compared to the control group while COP significantly decreased the protein expression levels of NLRP3 and ASC compared to those in the PI–IBS group [30].

#### 3.1.3. Chang-Kang-Fang Through TLR4/MyD88/NF-κB

The Toll-like receptors (TLR) family consists of innate immune pattern recognition receptors, with extracellular, transmembrane, and intracellular regions, primarily located on antigen-presenting and inflammatory cells. TLR4 recognizes pathogen-associated molecular patterns (PAMPs) through its leucine-rich repeats (LRRs) in the extracellular region. TLR4 triggers both MyD88-dependent and MyD88-independent signaling pathways. The MyD88-dependent pathway is further divided into two pathways: TLR4-MyD88/IL-1 Receptor-Associated Kinase (IRAK)-(MAPK) signaling pathway and the TLR4-MyD88/IRAK-Nuclear Factor Kappa B (NF-κB) Inducible Kinase (NIK)/NF-κB signaling pathway. The pathway involves MyD88 recruitment to TLR4, followed by assembly with tumor necrosis factor receptor-associated factor 6 (TRAF6), IRAK1, and IRAK4. Transforming Growth Factor-β-activated Kinase 1 (TAK1) helps phosphorylate the IκB kinase complex (IKK), leading to NF-κB nuclear translocation and activation of pro-inflammatory genes [28].

Chang-Kang-Fang (CKF), a multi-herb Chinese medicinal formula, has been used to treat IBS [44]. Zhang et al. investigated the potential mechanism of CKF for the treatment of diarrhea and visceral hypersensitivity in IBS-D Sprague–Dawley rats. Rats were randomly allocated into five groups: control group (sterilized distilled water), CKF-L (low dose of CKF, 0.54 g/kg/d, equivalent to the clinical dosage), CKF-M (middle dose of CKF, 1.08 g/kg/d), CKF-H (high dose of CKF, 2.15 g/kg/d), and TM group (Trimebutine maleate, positive control, 25 mg/kg/d). CKF significantly attenuated visceral hypersensitivity and improved symptoms of diarrhea. Furthermore, CKF treatment lowered IL-6, TNF-α, and IL-1β levels in the serum and colon tissues, while mitigating the symptoms of IBS-D rats by inhibiting the TLR4/NF-κB/NLRP3 pathway [28].

### 3.2. Synthetic Small Molecules

#### 3.2.1. BAY 11-7082 Through NF-κB Pathway

BAY 11-7082 (E)3-[(4-Methylphenyl)sulfonyl]-2-propene-nitrile is a sulfonic derivative that inhibits NF-κB by targeting the phosphorylation of inhibitor of nuclear factor kappa B (IκBα); the suppression of NF-κB eventually suppresses NLRP3 inflammasome activation [45]. Scuderi et al. evaluated the effect of BAY 11-7082 in a rat model with IBS-D. The animals were randomly assigned to six groups: Sham + vehicle (saline), Sham + BAY 11-7082 10 mg/kg, Sham + BAY 11-7082 30 mg/kg, IBS-D + vehicle (saline), IBS-D + BAY 11-7082 10 mg/kg and IBS-D + BAY 11-7082 30 mg/kg. The study measured myeloperoxidase (MPO) activity, an index of polymorphonuclear cell accumulation, and MDA, an indicator of lipid peroxidation (a molecular event involved in intestinal disorders). The results demonstrated that BAY 11-7082 at a dose of 30 mg/kg significantly reduced MPO, MDA, IL-1β, TNF-α, and IL-18 levels, as well as NF-κB and COX-2 expression. This research also showed that BAY 11-7082 significantly reduced edema, neutrophil infiltration, and loss of colonic epithelial structure [27].

#### 3.2.2. Tranilast Through ASC Oligomerization

Tranilast -N-[3,4-dimethoxycinnamoyl] anthranilic acid-, (TL) a tryptophan metabolite, was initially used to prevent the release of allergic reactions. TL also regulates the inflammatory response and inhibits the pro-inflammatory cytokines (IL-1β, IL-6, and TNF-α) expression in the serum [46].

TL directly binds to the NACHT domain of NLRP3 and inhibits the inflammasome assembly by blocking NLRP3 oligomerization. Consequently, caspase-1 activation and IL-1β production are inhibited without affecting ATPase activity, K+ efflux, or mitochondrial damage [13]. Nozu et al. evaluated the impact of TL on visceral hypersensitivity and colonic hyperpermeability induced by LPS or CRF in an IBS rat model. Both groups received intragastric administration of TL (20–200 mg/kg). To further investigate the role of the NLRP3 in the LPS model, the effects of β-hydroxybutyrate BHB were examined. The visceral pain threshold was measured by monitoring abdominal muscle contractions electrophysiologically. Colonic permeability was determined by quantifying the absorbed Evans blue within the colonic tissue. Colonic protein levels of NLRP3 and IL-1β were assessed by immunoblot. TL improved visceral pain and the colonic barrier while inhibiting LPS-induced NLRP3 and IL-1β expression. BHB also abolished visceral hypersensitivity and colonic hyperpermeability [6].

### 3.3. Organic Compounds

#### β-Hydroxybutyrate (BHB) Through ASC Oligomerization

BHB is one of the three ketone bodies, along with acetone and acetoacetate. Ketone bodies can act as immunomodulatory signaling metabolites [47,48,49]. Specifically, BHB reduces NLRP3 inflammasome-mediated IL-1β and IL-18 production in human monocytes [50]. BHB blocks NLRP3 inflammasome activation by preventing K^+^ efflux and regulating upstream events that reduce ASC speck formation. NLRP3 is oligomerized by the ATPase activity of its NACHT domain, which then recruits and oligomerizes ASC, leading to the caspase-1 activation and resulting in the IL-1β secretion. Treatment with BHB reduces ASC and activated-caspase-1 expression, resulting in the inhibition of IL-1β secretion. Nozu et al. first described BHB’s beneficial action in an IBS rat model, as described above [6,13,51].

### 3.4. Probiotics

#### *Bifidobacterium longum* Through TLR4/MyD88/NF-κB

Probiotic bacteria are live microorganisms, that, when administered in adequate amounts are effective for IBS symptom management [52]. Probiotics suppress NLRP3 inflammasome activation by binding to TLRs (2, 4, 9) via the MyD88-dependent signaling pathway, which inhibits NF-κB activation [53,54]. Infectious gastroenteritis is the strongest risk factor for the development of IBS with an incidence of PI_IBS 10% [55]. Fifty mice infected with *Trichinella spiralis* larvae were divided into five groups: 1. control group, 2. 2-week PI-IBS group, 3. 8-week control group, 4. 8-week PI-IBS group, and 5. 8-week PI-IBS group with *Bifidobacterium longum* intervention). The visceral sensitivity (measured with abdominal withdraw reflex scores) was significantly higher in the two post-infectious groups, and lower in the probiotic-treated group compared to the controls. IL-18 and IL-1β levels were also substantially higher in the two post-infectious groups compared to the controls but significantly lower in the PI-B group. Thus, investigators showed that *B. longum* may reduce visceral hypersensitivity in PI-IBS by down-regulating IL-18 and IL-1β and inhibiting NLRP3 [31].

## 4. Small Molecules, Phytochemicals, Organic Compounds, and Probiotics: Promising Therapeutic Choices for IBS

The NLRP3 inflammasome is related to multiple digestive disease pathogenesis, including inflammatory bowel diseases, non-alcoholic fatty liver disease, and gastric cancer. Inhibition of NLRP3-driven inflammation can be achieved directly or indirectly, through targeting signaling pathways, such as transcription and oligomerization, inhibition, or Gasdermin D cleavage inhibition [56].

Some studies highlighted the NLRP3 inflammasome’s role in regulating gut flora composition in mouse models [57,58]. X Yao et al. focused on the crosstalk complex between the NLRP3 and the gut microbiota and revealed that the hyperactive NLRP3 leads to an overproduction of IL-1β [59]. Probiotics have been proven to aid intestinal integrity and prevent microbial translocation [60]. Their ability to produce metabolic chemicals and antimicrobial substances inhibits the growth of other microorganisms competing for receptors and binding sites in the gastrointestinal mucosa [61]. *Bacillus coagulans* strain LBSC (DSM17654) is efficacious in alleviating IBS symptoms and improving the quality of life in IBS patients [62]. The underpinning protective functions of *Lactobacillus plantarum* in the amelioration of flatus were examined in 60 IBS patients for four weeks [63]. In another randomized, double-blind study in IBS patients, VSL#3 reduced flatulence and increased colonic transit time [64]. The efficacy of *Clostridium butyricum* in treating IBS-D was tested in a group of 200 patients for four weeks, improving symptoms overall, stool frequency, and quality of life [65]. *Bifidobacterium longum* and its metabolites can inhibit NLRP3 formation in macrophages associated with ulcerative colitis [66]. Research using probiotics is mentioned indicatively, as the thorough presentation of all studies is beyond the scope of this review. It is noteworthy that probiotics in IBS patients can modify the structure of the gut microbiota bringing about symptomatic relief and improving intestinal inflammation [52,66].

TL has been used to treat experimental colitis in rats, showing a protective anti-inflammatory effect, possibly due to the induction of the anti-inflammatory enzyme heme oxygenase-1 (HO-1) [67]. TL alleviated colonic damage and inflammation by activating the Nrf2-HO-1 pathway, in experimental colitis [68]. Nozu and colleagues investigated the intragastric administration of TL using an experimental IBS model, highlighting TL’s protective effects and its pharmacokinetic behaviors on visceral hypersensitivity and colonic hyperpermeability [6].

Studies indicated that BHB can be an important and instructive immune cell effector by inhibiting NLRP3 inflammasome activation and regulating intestinal pro-inflammatory Th17 cells [50,69]. In vivo, BHB attenuates the activation of caspase-1 and the secretion of IL-1β in mouse models of NLRP3-mediated diseases, such as Muckle–Wells Syndrome, Familial Cold, and IBS [6,50]. These anti-inflammatory effects of BHB via inhibiting the NLRP3 inflammasome suggest the potential of using treatments that increase circulating BHB levels to combat NLRP3-mediated pro-inflammatory diseases [50].

In one study, COP was found to mitigate dextran sulfate sodium (DSS)-induced ulcerative colitis in a murine model with significant amelioration in weight loss, disease activity index, intestinal permeability, and histologic alterations. Furthermore, COP downregulated the TXNIP, NLRP3, caspase-1, IL-1β, and IL-18 [70]. In vitro, COP significantly attenuated IL-1β secretion in macrophages stimulated with LPS plus ATP, nigericin, or monosodium urate crystal, by blocking caspase-1 activation [71]. COP prevented NLRP3 inflammasome assembly by affecting the binding between pro-caspase-1 and ASC and inhibited inflammasome priming by decreasing NLRP3 expression through the inactivation of the NF-κB pathway [43,71].

Li et al. evaluated the effectiveness of PF against DSS-induced colitis in mice and revealed that PF reduces tissue inflammation of the colon. PF suppressed NF-κB pathway activation, resulting in the regulation of this pro-inflammatory factor expression [72]. Wang et al. assessed the effects of PF on IBS in rats revealing that in the PF groups, the mucosal morphology of colon tissues was intact, and the glands were arranged neatly and structured clearly, without inflammatory cell infiltration. Compared to controls, PF groups had significantly higher pain thresholds and mRNA expression of ZO-1, IL-1β, TNF-α, and p-NF-κB levels, while serum levels of IL-1β, and TNF-α were reduced [73]. PF also significantly reduced diarrhea and abnormal bowel movements associated with abdominal pain and increased tight junction protein ZO-1 expression. In vitro, PF significantly regulated the IκBα gene and protein expression in an inflammatory Caco-2 cell model [72].

In IBS rat models, CKF normalized dysfunctions of combined chronic–acute stress induced in the central and peripheral nervous system and related to the differential levels of 5-hydroxytryptamine (5-HT) in the colon. The analysis of gut microbiota suggested that CKF could induce changes by decreasing Clostridiales, and the Firmicutes/Bacteroidetes ratio while increasing the levels of Lactobacillus [44]. Sun et al. elucidated the effectiveness of the CKF combined with a probiotic mixture (Pei-Fei-Kang, PFK) against IBS-D in rat models. They found that CKF with PFK may synergistically slow gastrointestinal motility and lower visceral hypersensitivity leading to a lower abdominal withdrawal reflex (AWR) score. Administering CKF and PFK significantly decreased 5-HT in the colon but increased it in the hippocampus. This intervention also improved intestinal barrier function by increasing ZO-1 and occludin levels in the colon and modulating gut microbiota composition [74].

BAY 11-7082, a selective NLRP3 inhibitor in macrophages, exhibited various pharmacological activities, including anticancer, neuroprotective, and anti-inflammatory effects [75,76]. Guruvaiah et al. successfully applied BAY 11-7082 to treat RAS oncogene mutant cancer cells [76], while Scuderi achieved reduced oral cancer progression [77,78]. In another study, BAY 11-7082 and its analogs demonstrated synergistic effects with penicillin G against methicillin-resistant *Staphylococcus aureus* exhibiting anti-inflammatory activity [79]. Recently, BAY 11-7082 prevented NLRP3 activation and reduced oxidative stress, improving mitochondrial health in diabetic rats [80]. These properties make BAY 11-7082 a promising molecule as a therapeutic target for IBS.

## 5. Conclusions

Extensive research and experiments are required to understand the exact role of NLRP3 inflammasome in IBS. To our knowledge, this is the first review of preclinical studies focusing on NLRP3 modulation in IBS. It summarizes the various activating factors of NLRP3 (miR-29a, ASC oligomerization, Nrf2, and TLR4/MyD88/NF-κB signaling pathways) and emphasizes its inhibitory mechanisms (TL, BHB, CKF, PF, COP, BAY 11-7082, and *Bifidobacterium longum*). Patients with IBS can benefit from probiotics in clinical practice, but the effectiveness of these probiotics varies by strain. Therefore, it is essential to choose the appropriate strain. Additionally, CKF, PF, COP, and BHB retain small bioactive components that provide new insights into how small molecules contribute to the therapeutic effects of IBS. However, the safety and delivery methods of miRNA inhibitors need to be evaluated in future dedicated trials. In conclusion, small molecules, probiotics, organic compounds, and phytochemical inhibitors targeting the NLRP3 inflammasome could have beneficial properties that merit proper clinical investigation for IBS symptoms management.

## Figures and Tables

**Table 1:** In vivo animal models.

Author, Year of Publication	Animal Model	Intervention	NLRP3/Mechanisms of Activity	Outcomes
Nozu et al., 2024 [6]	Rats with IBS	Tranilast	Inhibition through ASC oligomerization	Reduction in IL-1β, Inhibition of caspase-1 expression
		BHB		Inhibition of IL-1β production
Zhang et al., 2024 [28]	Rats with IBS-D	Chang-Kang-Fang	Inhibition, through TLR4/MyD88/NF-κB	Reduction in IL-1β, IL-6, TNF-α production
Ke et al., 2022 [29]	Mice with IBS-D	Paeoniflorin	Inhibition through miR-29a	Reduction in IL-1β, IL-18, TNF-α, MPO production
Xiong et al., 2022 [30]	Rats with PI-IBS	Coptisine	Inhibition through Nrf2 signaling	Reduction in IL-1β, IL-18, TNF-α production
Scuderi et al., 2020 [27]	Rats with IBS-D	BAY 11-7082	Inhibition through NF-κB pathway	Reduction in IL-1β, IL-18, TNF-α, MPO, MDA, COX-2. Production Reestablishment of NF-κB/Iκb-α expression
Gu et al., 2016 [31]	Mice with PI-IBS	*Bifidobacterium longum*	Inhibition TLR4/MyD88/NF-κB	Reduction in IL-1β, IL-18 production

ASC: apoptosis-associated speck-like protein; BHB: β-hydroxybutyrate; COX-2: Cyclooxygenase-2; IBS-D: IBS with diarrhea subtype; interleukin (IL)-1β; MyD88: Myeloid Differentiation Factor 88; MDA: malondialdehyde; MPO: myeloperoxidase; PI-IBS: post-infectious irritable bowel syndrome; TNF-α: Tumor necrosis factor alpha.

## Data Availability

Not applicable.

## Abbreviations

5-HT	5-hydroxytryptamine
AWR	Abdominal withdrawal reflex
ASC	Apoptosis-associated speck-like protein
BHB	β-hydroxybutyrate
CARD	Caspase recruitment domain
CKF	Chang-Kang-Fang
COX-2	Cyclooxygenase-2
COP	Coptisine
CRF	Corticotropin-releasing factor
DAMPs	Damage-associated molecular patterns
DSS	Dextran sulfate sodium
ENS	Enteric nervous system
GSDMD	Gasdermin D
HO-1	Heme oxygenase-1
IBS-C	Constipation-predominant irritable bowel syndrome
IBS-D	Diarrhea-predominant irritable bowel syndrome
IECs	Intestinal epithelial cells
IκBα	Inhibitor of nuclear factor kappa B
IKK	IκB kinase complex
IL-1β	Interleukin-1β
IL-6	Interleukin-6
IRAK	IL-1 Receptor-Associated Kinase
LPS	Lipopolysaccharide
LRRs	Leucine-rich repeats
MAPK	Mitogen-Activated Protein Kinase
MCs	Mast cells
MDA	Malondialdehyde
miRNAs	MicroRNAs
MPO	Myeloperoxidase
MyD88	Myeloid Differentiation Factor 88 NC: Normal controls
NF-κB	Nuclear factor kappa-B
NLR	NOD-like receptor
NLRP3	NLR family pyrin domain-containing protein 3
NMS	Neonatal maternal separation
Nrf2	Transcription factor nuclear factor erythroid 2-related factor 2
PAMPs	Pathogen-associated molecular patterns
PBMCs	Peripheral blood mononuclear cells
PFK	Pei-Fei-Kang
PF	Paeoniflorin
PGE2	Prostaglandin E2
PI-IBS	Post-infectious irritable bowel syndrome
PYD	Pyrin domain
ROS	Reactive oxygen species
RS	Restraint stress TAK1: Transforming Growth Factor-β-activated Kinase 1
TIR	Toll/IL-1 receptor
TL	Tranilast
TLR4	Toll-like receptor 4
TNF α	Tumor necrosis factor-alpha
TRAF6	Tumor necrosis factor receptor-associated factor 6
TRX	Thioredoxin
TXNIP	Thioredoxin-interacting protein
ZO-1	Zonulin protein
